# 404. Impact of cross-coronavirus humoral immunity in post-acute sequelae of COVID-19 in systemic autoimmune rheumatic diseases patients

**DOI:** 10.1093/ofid/ofad500.474

**Published:** 2023-11-27

**Authors:** Jonathan Herman, Caroline Atyeo, Yonatan Zur, Claire Cook, Naomi Patel, Kathleen Vanni, Emily Kowalski, Grace Qian, Shruthi Srivatsan, Nancy Shadick, Deepak Rao, Benjamin Kellman, Colin Mann, Douglas A Lauffenburger, Zachary Wallace, Jeffrey Sparks, Galit Alter

**Affiliations:** Brigham and Women's Hospital / Ragon Institute, Cambridge, Massachusetts; Ragon Institute, Cambirdge, Massachusetts; Ragon Institute, Cambirdge, Massachusetts; Massachusetts General Hospital, Cambridge, Massachusetts; Massachusetts General Hospital, Cambridge, Massachusetts; Brigham and Women's Hospital / Ragon Institute, Cambridge, Massachusetts; Brigham and Women's Hospital / Ragon Institute, Cambridge, Massachusetts; Brigham and Women's Hospital / Ragon Institute, Cambridge, Massachusetts; Massachusetts General Hospital, Cambridge, Massachusetts; Brigham and Women's Hospital / Ragon Institute, Cambridge, Massachusetts; Brigham and Women's Hospital / Ragon Institute, Cambridge, Massachusetts; Ragon Institute, Cambirdge, Massachusetts; Ragon Institute, Cambirdge, Massachusetts; Massachusetts Institute of Technology, Cambridge, Massachusetts; Massachusetts General Hospital, Cambridge, Massachusetts; Brigham and Women's Hospital, Cambridge, Massachusetts; Ragon Institute of MGH, MIT, and Harvard, Cambridge, Massachusetts

## Abstract

**Background:**

Beyond the acute illness caused by SARS-CoV-2, about one-fifth of infections unpredictably result in long-term persistence of symptoms despite the apparent clearance of infection. Insights into the mechanisms that underlie post-acute sequelae of COVID-19 (PASC) will be critical for the prevention and clinical management of long-term complications of COVID-19. Several hypotheses have been proposed that may account for the development of PASC, including persistence of virus or the dysregulation of immunity. Among the immunological changes noted in PASC, alterations in humoral immunity have been observed in some patient subsets.

**Methods:**

To begin to determine whether SARS-CoV-2 or other pathogen specific humoral immune responses evolve uniquely in PASC, we performed comprehensive antibody profiling against SARS-CoV-2, a panel of endemic pathogens, and a panel of routine vaccine antigens using Systems Serology in two independent cohorts of patients with pre-existing systemic autoimmune rheumatic disease (SARD) who either developed or did not develop PASC.

**Results:**

A distinct qualitative shift observed in Fcγ receptor binding was observed in individuals with PASC. Specifically, individuals with PASC harbored weaker Fcγ receptor binding anti-SARS-CoV-2 antibodies and a significantly stronger Fcγ receptor binding antibody response against endemic Coronavirus OC43. Individuals with PASC, further, generated more avid IgM responses and developed an OC43 S2-specific antibody response with stronger Fcγ-receptor binding, linked to cross reactivity across SARS-CoV-2 and common coronaviruses.

**Conclusion:**

These findings from two independent cohorts implicate previous common Coronavirus imprinting as a marker for the development of PASC in individuals with SARDs.

**Disclosures:**

**Douglas A. Lauffenburger, PhD**, Sanofi: Board Member|Sanofi: Honoraria|Sanofi: Honoraria **Zachary Wallace, MD, MSc**, Bristol-Myers Squibb: Grant/Research Support|Horizon: Advisor/Consultant|MedPace: Advisor/Consultant|Principia/Sanofi: Grant/Research Support|Sanofi: Advisor/Consultant|Shionogi: Advisor/Consultant|Viela Bio: Advisor/Consultant|Zenas BioPharma: Advisor/Consultant **Jeffrey Sparks, MD**, AbbVie: Advisor/Consultant|Amgen: Advisor/Consultant|Boehringer Ingelheim: Advisor/Consultant|Bristol Myers Squibb: Advisor/Consultant|Bristol Myers Squibb: Grant/Research Support|Gilead: Advisor/Consultant|Inova Diagnostics: Advisor/Consultant|Janssen: Advisor/Consultant|Optum: Advisor/Consultant|Pfizer: Advisor/Consultant **Galit Alter, PhD**, Leyden Labs: Ownership Interest|Moderna Therapeutics: Employee|Seromyx Systems: Ownership Interest

